# Supplementary material for: Repetitive transcranial magnetic stimulation activates glial cells and inhibits neurogenesis after pneumococcal meningitis
Source: PLoS One. 2020 Sep 11;15(9):e0232863. doi: 10.1371/journal.pone.0232863 (PMC7485822; doi:10.1371/journal.pone.0232863)
Supplement: S1 Fig — Bacterial CSF titers were comparable between the three different groups cTBS, iTBS and sham and indicated a comparable severity of infection (A). Development of relative weight after infection further proved comparability between different groups, as PM-induced weight loss and recovery was non-different within all analysed animals analysed by 2-way ANOVA (B). Statistical differences for bacterial titres were assessed using one-way ANOVA with Tukey’s multiple comparison test to adjust for multiple testing. (PDF) [file pone.0232863.s001.pdf]

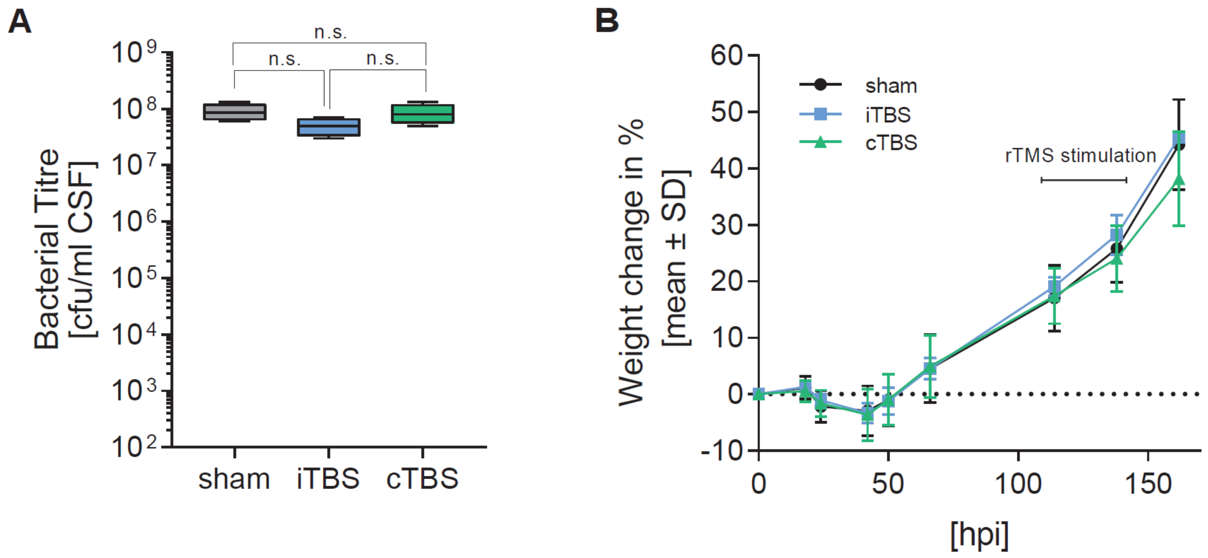


**Figure S1. Bacterial CSF titres and weight development during pneumococcal meningitis.** Bacterial CSF titres were comparable between the three different groups cTBS, iTBS and sham and indicated a comparable severity of infection (A). Development of relative weight after infection further proved comparability between different groups, as PM-induced weight loss and recovery was non-different within all analysed animals analysed by 2-way ANOVA (B). Statistical differences for bacterial titres were assessed using one-way ANOVA with Tukey's multiple comparison test to adjust for multiple testing.
