# Supplementary material for: Repetitive transcranial magnetic stimulation activates glial cells and inhibits neurogenesis after pneumococcal meningitis
Source: PLoS One. 2020 Sep 11;15(9):e0232863. doi: 10.1371/journal.pone.0232863 (PMC7485822; doi:10.1371/journal.pone.0232863)
Supplement: S6 Fig — (A) Astroglial cells–containing astrocytes (GFAP), microglia and oligodendrocytes (not shown)–isolated from neonatal rat brains were kept in culture for 2 weeks before stimulation. (B) Stimulation with 4 trains of cTBS on two consecutive days increased cytokine release. Significantly increased release of IL-1β, IL-10 and TNF-α was found after stimulation with cTBS, which also increased levels of IL-6 but only with a statistical trend (p = 0.104). An unpaired Student t test was used to assess statistical differences between cTBS-stimulated and control cell cultures. (PDF) [file pone.0232863.s006.pdf]

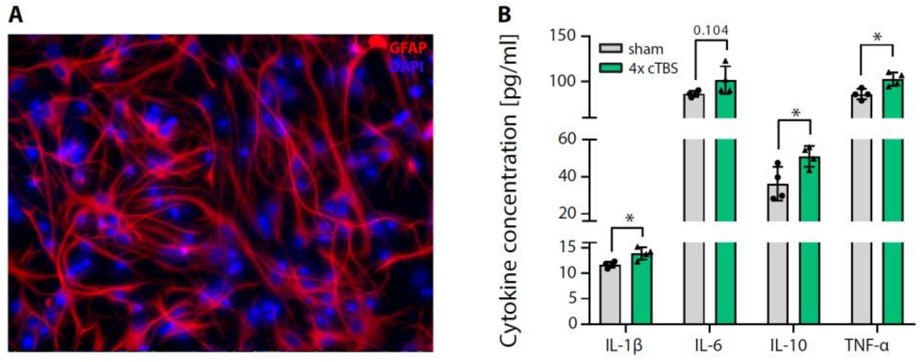

Figure S6. In vitro stimulation of rat astroglial cell cultures by cTBS. (**A**) Astroglial cells – containing astrocytes (GFAP), microglia and oligodendrocytes (not shown) – isolated from neonatal rat brains were kept in culture for 2 weeks before stimulation. (**B**) Stimulation with 4 trains of cTBS on two consecutive days increased cytokine release. Significantly increased release of IL-1β, IL-10 and TNF-α was found after stimulation with cTBS, which also increased levels of IL-6 but only with a statistical trend ($p = 0.104$).
