# Supplementary material for: Repetitive transcranial magnetic stimulation activates glial cells and inhibits neurogenesis after pneumococcal meningitis
Source: PLoS One. 2020 Sep 11;15(9):e0232863. doi: 10.1371/journal.pone.0232863 (PMC7485822; doi:10.1371/journal.pone.0232863)
Supplement: S1 Table — (DOCX) [file pone.0232863.s007.docx]

Table S1. Overrepresented gene ontologies of downregulated genes after cTBS in the cortex.

| GO term | Description | p-value | FDR q-value |
| --- | --- | --- | --- |
| GO:0050804 | modulation of chemical synaptic transmission | 1.03E-23 | 1.59E-19 |
| GO:0099177 | regulation of trans-synaptic signaling | 1.17E-23 | 9.06E-20 |
| GO:0010975 | regulation of neuron projection development | 5.02E-18 | 2.59E-14 |
| GO:0051960 | regulation of nervous system development | 2.17E-17 | 8.39E-14 |
| GO:0120035 | regulation of plasma membrane bounded cell projection organization | 1.15E-16 | 3.55E-13 |
| GO:0031344 | regulation of cell projection organization | 2.53E-16 | 6.53E-13 |
| GO:0042391 | regulation of membrane potential | 2.78E-16 | 6.13E-13 |
| GO:0045664 | regulation of neuron differentiation | 6.94E-16 | 1.34E-12 |
| GO:0050767 | regulation of neurogenesis | 9.6E-16 | 1.65E-12 |
| GO:0060284 | regulation of cell development | 1.44E-14 | 2.23E-11 |
| GO:0010769 | regulation of cell morphogenesis involved in differentiation | 4.81E-14 | 6.76E-11 |
| GO:0050808 | synapse organization | 7.93E-14 | 1.02E-10 |
| GO:0032879 | regulation of localization | 2.33E-13 | 2.77E-10 |
| GO:0050890 | cognition | 4.33E-13 | 4.78E-10 |
| GO:0017158 | regulation of calcium ion-dependent exocytosis | 8.44E-13 | 8.7E-10 |
| GO:0007612 | learning | 1.59E-12 | 1.54E-9 |
| GO:0022604 | regulation of cell morphogenesis | 2.82E-12 | 2.57E-9 |
| GO:2000463 | positive regulation of excitatory postsynaptic potential | 3.26E-12 | 2.8E-9 |
| GO:0007611 | learning or memory | 7.61E-12 | 6.19E-9 |
| GO:0050789 | regulation of biological process | 1.03E-11 | 7.92E-9 |
| GO:0050806 | positive regulation of synaptic transmission | 1.28E-11 | 9.43E-9 |
| GO:0050770 | regulation of axonogenesis | 1.76E-11 | 1.24E-8 |
| GO:0098693 | regulation of synaptic vesicle cycle | 1.84E-11 | 1.23E-8 |
| GO:0048858 | cell projection morphogenesis | 2.11E-11 | 1.36E-8 |
| GO:0048812 | neuron projection morphogenesis | 2.96E-11 | 1.83E-8 |
| GO:0050794 | regulation of cellular process | 3.63E-11 | 2.16E-8 |
| GO:0065007 | biological regulation | 4.2E-11 | 2.4E-8 |
| GO:0120039 | plasma membrane bounded cell projection morphogenesis | 6.33E-11 | 3.49E-8 |
| GO:0051049 | regulation of transport | 6.46E-11 | 3.44E-8 |
| GO:0046928 | regulation of neurotransmitter secretion | 8.16E-11 | 4.2E-8 |
| GO:0032990 | cell part morphogenesis | 9.22E-11 | 4.6E-8 |
| GO:0051128 | regulation of cellular component organization | 1.03E-10 | 4.96E-8 |
| GO:0098815 | modulation of excitatory postsynaptic potential | 1.16E-10 | 5.42E-8 |
| GO:0051588 | regulation of neurotransmitter transport | 1.38E-10 | 6.29E-8 |
| GO:0048167 | regulation of synaptic plasticity | 1.87E-10 | 8.27E-8 |
| GO:0007610 | behavior | 2.91E-10 | 1.25E-7 |
| GO:0051179 | localization | 3.25E-10 | 1.36E-7 |
| GO:0031644 | regulation of neurological system process | 3.29E-10 | 1.34E-7 |
| GO:1903305 | regulation of regulated secretory pathway | 3.39E-10 | 1.34E-7 |
| GO:0060341 | regulation of cellular localization | 3.62E-10 | 1.4E-7 |
| GO:0043269 | regulation of ion transport | 3.9E-10 | 1.47E-7 |
| GO:0034765 | regulation of ion transmembrane transport | 4.77E-10 | 1.75E-7 |
| GO:0030030 | cell projection organization | 5.24E-10 | 1.88E-7 |
| GO:0030001 | metal ion transport | 6.13E-10 | 2.15E-7 |
| GO:0099003 | vesicle-mediated transport in synapse | 6.33E-10 | 2.17E-7 |
| GO:0098662 | inorganic cation transmembrane transport | 7.01E-10 | 2.35E-7 |
| GO:0050877 | nervous system process | 7.08E-10 | 2.33E-7 |
| GO:0099643 | signal release from synapse | 7.48E-10 | 2.41E-7 |
| GO:0050807 | regulation of synapse organization | 1.4E-9 | 4.41E-7 |
| GO:2000026 | regulation of multicellular organismal development | 1.46E-9 | 4.52E-7 |
| GO:0031646 | positive regulation of neurological system process | 2.68E-9 | 8.11E-7 |
| GO:0099084 | postsynaptic specialization organization | 3.22E-9 | 9.58E-7 |
| GO:0050905 | neuromuscular process | 4.02E-9 | 1.17E-6 |
| GO:2000300 | regulation of synaptic vesicle exocytosis | 4.87E-9 | 1.39E-6 |
| GO:0006810 | transport | 6.44E-9 | 1.81E-6 |
| GO:0045595 | regulation of cell differentiation | 7.63E-9 | 2.11E-6 |
| GO:0031346 | positive regulation of cell projection organization | 7.66E-9 | 2.08E-6 |
| GO:0098660 | inorganic ion transmembrane transport | 7.92E-9 | 2.11E-6 |
| GO:0051234 | establishment of localization | 8.98E-9 | 2.35E-6 |
| GO:1902803 | regulation of synaptic vesicle transport | 1.03E-8 | 2.65E-6 |
| GO:0098655 | cation transmembrane transport | 1.37E-8 | 3.46E-6 |
| GO:0034762 | regulation of transmembrane transport | 1.57E-8 | 3.91E-6 |
| GO:0017156 | calcium ion regulated exocytosis | 1.67E-8 | 4.11E-6 |
| GO:0017157 | regulation of exocytosis | 2.46E-8 | 5.94E-6 |
| GO:0051962 | positive regulation of nervous system development | 2.54E-8 | 6.04E-6 |
| GO:0048489 | synaptic vesicle transport | 2.89E-8 | 6.76E-6 |
| GO:0097480 | establishment of synaptic vesicle localization | 2.89E-8 | 6.66E-6 |
| GO:0051650 | establishment of vesicle localization | 2.94E-8 | 6.69E-6 |
| GO:0071805 | potassium ion transmembrane transport | 3.28E-8 | 7.35E-6 |
| GO:0071804 | cellular potassium ion transport | 3.28E-8 | 7.25E-6 |
| GO:0007613 | memory | 3.28E-8 | 7.14E-6 |
| GO:0045666 | positive regulation of neuron differentiation | 3.51E-8 | 7.54E-6 |
| GO:0010976 | positive regulation of neuron projection development | 3.65E-8 | 7.72E-6 |
| GO:0060627 | regulation of vesicle-mediated transport | 3.74E-8 | 7.82E-6 |
| GO:0016079 | synaptic vesicle exocytosis | 4.76E-8 | 9.8E-6 |
| GO:0006813 | potassium ion transport | 4.81E-8 | 9.79E-6 |
| GO:0051239 | regulation of multicellular organismal process | 4.99E-8 | 1E-5 |
| GO:0031345 | negative regulation of cell projection organization | 6.79E-8 | 1.35E-5 |
| GO:0099601 | regulation of neurotransmitter receptor activity | 7.3E-8 | 1.43E-5 |
| GO:0010646 | regulation of cell communication | 8.42E-8 | 1.63E-5 |
| GO:0035176 | social behavior | 9.4E-8 | 1.79E-5 |
| GO:0051703 | intraspecies interaction between organisms | 9.4E-8 | 1.77E-5 |
| GO:0003008 | system process | 1.06E-7 | 1.97E-5 |
| GO:0016192 | vesicle-mediated transport | 1.07E-7 | 1.97E-5 |
| GO:0097479 | synaptic vesicle localization | 1.09E-7 | 1.98E-5 |
| GO:0023052 | signaling | 1.18E-7 | 2.12E-5 |
| GO:0001505 | regulation of neurotransmitter levels | 1.22E-7 | 2.17E-5 |
| GO:0023051 | regulation of signaling | 1.31E-7 | 2.31E-5 |
| GO:0050773 | regulation of dendrite development | 1.39E-7 | 2.41E-5 |
| GO:0050793 | regulation of developmental process | 1.54E-7 | 2.65E-5 |
| GO:0050769 | positive regulation of neurogenesis | 1.58E-7 | 2.68E-5 |
| GO:0007409 | axonogenesis | 1.89E-7 | 3.17E-5 |
| GO:0045956 | positive regulation of calcium ion-dependent exocytosis | 2.04E-7 | 3.39E-5 |
| GO:0051648 | vesicle localization | 2.23E-7 | 3.67E-5 |
| GO:0006812 | cation transport | 2.56E-7 | 4.17E-5 |
| GO:0031175 | neuron projection development | 3.14E-7 | 5.05E-5 |
| GO:0010977 | negative regulation of neuron projection development | 3.57E-7 | 5.68E-5 |
| GO:0022603 | regulation of anatomical structure morphogenesis | 3.57E-7 | 5.63E-5 |
| GO:0035418 | protein localization to synapse | 3.98E-7 | 6.21E-5 |
| GO:0070588 | calcium ion transmembrane transport | 4E-7 | 6.19E-5 |
| GO:0032989 | cellular component morphogenesis | 4.67E-7 | 7.15E-5 |
| GO:0061387 | regulation of extent of cell growth | 6.18E-7 | 9.36E-5 |
| GO:0023061 | signal release | 6.23E-7 | 9.35E-5 |
| GO:0034220 | ion transmembrane transport | 6.42E-7 | 9.54E-5 |
| GO:1903530 | regulation of secretion by cell | 7.8E-7 | 1.15E-4 |
| GO:0048640 | negative regulation of developmental growth | 8.14E-7 | 1.19E-4 |
| GO:0048814 | regulation of dendrite morphogenesis | 8.42E-7 | 1.22E-4 |
| GO:0051963 | regulation of synapse assembly | 9.79E-7 | 1.4E-4 |
| GO:0045055 | regulated exocytosis | 1.11E-6 | 1.58E-4 |
| GO:0006811 | ion transport | 1.15E-6 | 1.61E-4 |
| GO:0051668 | localization within membrane | 1.39E-6 | 1.93E-4 |
| GO:0006836 | neurotransmitter transport | 1.58E-6 | 2.18E-4 |
| GO:0006816 | calcium ion transport | 1.58E-6 | 2.16E-4 |
| GO:0010720 | positive regulation of cell development | 1.61E-6 | 2.19E-4 |
| GO:0097106 | postsynaptic density organization | 1.63E-6 | 2.19E-4 |
| GO:2001257 | regulation of cation channel activity | 1.65E-6 | 2.19E-4 |
| GO:0010771 | negative regulation of cell morphogenesis involved in differentiation | 1.77E-6 | 2.33E-4 |
| GO:0051046 | regulation of secretion | 2.02E-6 | 2.64E-4 |
| GO:0098916 | anterograde trans-synaptic signaling | 2.08E-6 | 2.71E-4 |
| GO:0007268 | chemical synaptic transmission | 2.08E-6 | 2.68E-4 |
| GO:0048638 | regulation of developmental growth | 2.25E-6 | 2.87E-4 |
| GO:0051966 | regulation of synaptic transmission, glutamatergic | 2.3E-6 | 2.91E-4 |
| GO:0065008 | regulation of biological quality | 2.34E-6 | 2.93E-4 |
| GO:0008306 | associative learning | 2.75E-6 | 3.43E-4 |
| GO:0032412 | regulation of ion transmembrane transporter activity | 2.99E-6 | 3.69E-4 |
| GO:0097120 | receptor localization to synapse | 3.32E-6 | 4.07E-4 |
| GO:0044087 | regulation of cellular component biogenesis | 3.79E-6 | 4.61E-4 |
| GO:0050885 | neuromuscular process controlling balance | 3.9E-6 | 4.71E-4 |
| GO:0055085 | transmembrane transport | 3.98E-6 | 4.77E-4 |
| GO:0015672 | monovalent inorganic cation transport | 4E-6 | 4.76E-4 |
| GO:0099558 | maintenance of synapse structure | 4.01E-6 | 4.73E-4 |
| GO:0099175 | regulation of postsynapse organization | 4.54E-6 | 5.31E-4 |
| GO:0044057 | regulation of system process | 4.56E-6 | 5.3E-4 |
| GO:0051705 | multi-organism behavior | 4.63E-6 | 5.34E-4 |
| GO:0098880 | maintenance of postsynaptic specialization structure | 5.52E-6 | 6.31E-4 |
| GO:0030516 | regulation of axon extension | 5.53E-6 | 6.28E-4 |
| GO:0099536 | synaptic signaling | 5.68E-6 | 6.4E-4 |
| GO:0099537 | trans-synaptic signaling | 5.68E-6 | 6.36E-4 |
| GO:0051056 | regulation of small GTPase mediated signal transduction | 5.68E-6 | 6.31E-4 |
| GO:0099072 | regulation of postsynaptic membrane neurotransmitter receptor levels | 5.83E-6 | 6.43E-4 |
| GO:0022898 | regulation of transmembrane transporter activity | 5.95E-6 | 6.52E-4 |
| GO:0051129 | negative regulation of cellular component organization | 6E-6 | 6.54E-4 |
| GO:0072657 | protein localization to membrane | 6.31E-6 | 6.82E-4 |
| GO:0050768 | negative regulation of neurogenesis | 6.69E-6 | 7.18E-4 |
| GO:0050771 | negative regulation of axonogenesis | 6.95E-6 | 7.41E-4 |
| GO:0010721 | negative regulation of cell development | 7.01E-6 | 7.42E-4 |
| GO:1902667 | regulation of axon guidance | 8.89E-6 | 9.35E-4 |
| GO:1900006 | positive regulation of dendrite development | 1E-5 | 1.05E-3 |
| GO:0007416 | synapse assembly | 1.03E-5 | 1.07E-3 |
| GO:0051961 | negative regulation of nervous system development | 1.12E-5 | 1.16E-3 |
| GO:0006887 | exocytosis | 1.21E-5 | 1.24E-3 |
| GO:0032409 | regulation of transporter activity | 1.24E-5 | 1.26E-3 |
| GO:2000311 | regulation of AMPA receptor activity | 1.27E-5 | 1.28E-3 |
| GO:0086010 | membrane depolarization during action potential | 1.27E-5 | 1.27E-3 |
| GO:1903539 | protein localization to postsynaptic membrane | 1.29E-5 | 1.29E-3 |
| GO:0070509 | calcium ion import | 1.44E-5 | 1.43E-3 |
| GO:0098698 | postsynaptic specialization assembly | 1.46E-5 | 1.44E-3 |
| GO:0008542 | visual learning | 1.47E-5 | 1.44E-3 |
| GO:0016310 | phosphorylation | 1.68E-5 | 1.64E-3 |
| GO:0032940 | secretion by cell | 1.9E-5 | 1.84E-3 |
| GO:0007632 | visual behavior | 2.08E-5 | 1.99E-3 |
| GO:0007267 | cell-cell signaling | 2.1E-5 | 2E-3 |
| GO:0070838 | divalent metal ion transport | 2.1E-5 | 2E-3 |
| GO:0007269 | neurotransmitter secretion | 2.33E-5 | 2.19E-3 |
| GO:0051649 | establishment of localization in cell | 2.47E-5 | 2.32E-3 |
| GO:0072511 | divalent inorganic cation transport | 2.61E-5 | 2.43E-3 |
| GO:1903307 | positive regulation of regulated secretory pathway | 3.13E-5 | 2.9E-3 |
| GO:0043954 | cellular component maintenance | 3.18E-5 | 2.92E-3 |
| GO:0051656 | establishment of organelle localization | 3.79E-5 | 3.47E-3 |
| GO:0001508 | action potential | 3.96E-5 | 3.6E-3 |
| GO:0051641 | cellular localization | 4E-5 | 3.61E-3 |
| GO:0007626 | locomotory behavior | 4.06E-5 | 3.65E-3 |
| GO:0045665 | negative regulation of neuron differentiation | 4.13E-5 | 3.69E-3 |
| GO:0051899 | membrane depolarization | 4.5E-5 | 4E-3 |
| GO:0060998 | regulation of dendritic spine development | 4.56E-5 | 4.02E-3 |
| GO:0048168 | regulation of neuronal synaptic plasticity | 4.62E-5 | 4.06E-3 |
| GO:0051640 | organelle localization | 4.91E-5 | 4.29E-3 |
| GO:0098962 | regulation of postsynaptic neurotransmitter receptor activity | 4.95E-5 | 4.29E-3 |
| GO:0098609 | cell-cell adhesion | 5.27E-5 | 4.55E-3 |
| GO:0140029 | exocytic process | 5.37E-5 | 4.61E-3 |
| GO:0051130 | positive regulation of cellular component organization | 5.39E-5 | 4.6E-3 |
| GO:0045185 | maintenance of protein location | 5.57E-5 | 4.73E-3 |
| GO:0043547 | positive regulation of GTPase activity | 5.84E-5 | 4.93E-3 |
| GO:0070050 | neuron cellular homeostasis | 6.11E-5 | 5.13E-3 |
| GO:0032507 | maintenance of protein location in cell | 6.13E-5 | 5.12E-3 |
| GO:0030534 | adult behavior | 6.72E-5 | 5.58E-3 |
| GO:0046578 | regulation of Ras protein signal transduction | 7.02E-5 | 5.8E-3 |
| GO:0045921 | positive regulation of exocytosis | 7.15E-5 | 5.88E-3 |
| GO:0046834 | lipid phosphorylation | 7.93E-5 | 6.48E-3 |
| GO:1900449 | regulation of glutamate receptor signaling pathway | 8.21E-5 | 6.68E-3 |
| GO:0120036 | plasma membrane bounded cell projection organization | 8.29E-5 | 6.71E-3 |
| GO:0043087 | regulation of GTPase activity | 9.73E-5 | 7.83E-3 |
| GO:0030517 | negative regulation of axon extension | 9.91E-5 | 7.94E-3 |
| GO:0007215 | glutamate receptor signaling pathway | 9.91E-5 | 7.9E-3 |
| GO:0001558 | regulation of cell growth | 1E-4 | 7.96E-3 |
| GO:0007154 | cell communication | 1.05E-4 | 8.27E-3 |
| GO:0032386 | regulation of intracellular transport | 1.05E-4 | 8.26E-3 |
| GO:0099562 | maintenance of postsynaptic density structure | 1.06E-4 | 8.29E-3 |
| GO:2001259 | positive regulation of cation channel activity | 1.13E-4 | 8.8E-3 |
| GO:0040013 | negative regulation of locomotion | 1.14E-4 | 8.81E-3 |
| GO:0035303 | regulation of dephosphorylation | 1.17E-4 | 8.96E-3 |
| GO:0051952 | regulation of amine transport | 1.25E-4 | 9.55E-3 |
| GO:0060078 | regulation of postsynaptic membrane potential | 1.29E-4 | 9.84E-3 |
| GO:0106027 | neuron projection organization | 1.44E-4 | 1.09E-2 |
| GO:0032012 | regulation of ARF protein signal transduction | 1.61E-4 | 1.22E-2 |
| GO:0043270 | positive regulation of ion transport | 1.63E-4 | 1.22E-2 |
| GO:0046903 | secretion | 1.74E-4 | 1.3E-2 |
| GO:0015812 | gamma-aminobutyric acid transport | 1.75E-4 | 1.3E-2 |
| GO:0086012 | membrane depolarization during cardiac muscle cell action potential | 1.75E-4 | 1.29E-2 |
| GO:0099173 | postsynapse organization | 1.75E-4 | 1.29E-2 |
| GO:0006904 | vesicle docking involved in exocytosis | 1.75E-4 | 1.28E-2 |
| GO:1990504 | dense core granule exocytosis | 1.86E-4 | 1.35E-2 |
| GO:0014051 | gamma-aminobutyric acid secretion | 1.86E-4 | 1.35E-2 |
| GO:0007165 | signal transduction | 1.91E-4 | 1.38E-2 |
| GO:0016043 | cellular component organization | 1.94E-4 | 1.39E-2 |
| GO:0048841 | regulation of axon extension involved in axon guidance | 2.02E-4 | 1.44E-2 |
| GO:0060079 | excitatory postsynaptic potential | 2.06E-4 | 1.47E-2 |
| GO:0046580 | negative regulation of Ras protein signal transduction | 2.36E-4 | 1.67E-2 |
| GO:1990138 | neuron projection extension | 2.39E-4 | 1.69E-2 |
| GO:0035637 | multicellular organismal signaling | 2.39E-4 | 1.68E-2 |
| GO:1904062 | regulation of cation transmembrane transport | 2.56E-4 | 1.79E-2 |
| GO:0086002 | cardiac muscle cell action potential involved in contraction | 2.65E-4 | 1.85E-2 |
| GO:0021819 | layer formation in cerebral cortex | 2.72E-4 | 1.89E-2 |
| GO:0061001 | regulation of dendritic spine morphogenesis | 3.05E-4 | 2.1E-2 |
| GO:1904031 | positive regulation of cyclin-dependent protein kinase activity | 3.08E-4 | 2.12E-2 |
| GO:0099632 | protein transport within plasma membrane | 3.09E-4 | 2.12E-2 |
| GO:0099637 | neurotransmitter receptor transport | 3.09E-4 | 2.11E-2 |
| GO:0007420 | brain development | 3.11E-4 | 2.11E-2 |
| GO:0060999 | positive regulation of dendritic spine development | 3.24E-4 | 2.19E-2 |
| GO:0008104 | protein localization | 3.3E-4 | 2.22E-2 |
| GO:0032880 | regulation of protein localization | 3.32E-4 | 2.22E-2 |
| GO:0001956 | positive regulation of neurotransmitter secretion | 3.39E-4 | 2.26E-2 |
| GO:0071840 | cellular component organization or biogenesis | 3.49E-4 | 2.31E-2 |
| GO:0065009 | regulation of molecular function | 3.5E-4 | 2.31E-2 |
| GO:0033036 | macromolecule localization | 3.51E-4 | 2.31E-2 |
| GO:0090129 | positive regulation of synapse maturation | 3.57E-4 | 2.34E-2 |
| GO:0046958 | nonassociative learning | 3.57E-4 | 2.33E-2 |
| GO:0010770 | positive regulation of cell morphogenesis involved in differentiation | 3.67E-4 | 2.38E-2 |
| GO:0051968 | positive regulation of synaptic transmission, glutamatergic | 3.76E-4 | 2.43E-2 |
| GO:0034613 | cellular protein localization | 3.82E-4 | 2.46E-2 |
| GO:0045926 | negative regulation of growth | 3.97E-4 | 2.54E-2 |
| GO:0045762 | positive regulation of adenylate cyclase activity | 4.06E-4 | 2.59E-2 |
| GO:0016081 | synaptic vesicle docking | 4.06E-4 | 2.58E-2 |
| GO:0090128 | regulation of synapse maturation | 4.06E-4 | 2.57E-2 |
| GO:1903827 | regulation of cellular protein localization | 4.08E-4 | 2.58E-2 |
| GO:0140056 | organelle localization by membrane tethering | 4.17E-4 | 2.62E-2 |
| GO:1904064 | positive regulation of cation transmembrane transport | 4.17E-4 | 2.61E-2 |
| GO:0045597 | positive regulation of cell differentiation | 4.34E-4 | 2.7E-2 |
| GO:0032414 | positive regulation of ion transmembrane transporter activity | 4.46E-4 | 2.77E-2 |
| GO:0040008 | regulation of growth | 4.53E-4 | 2.8E-2 |
| GO:0034767 | positive regulation of ion transmembrane transport | 4.6E-4 | 2.83E-2 |
| GO:0070727 | cellular macromolecule localization | 4.92E-4 | 3.02E-2 |
| GO:0007010 | cytoskeleton organization | 4.97E-4 | 3.04E-2 |
| GO:0051493 | regulation of cytoskeleton organization | 5.05E-4 | 3.07E-2 |
| GO:0048278 | vesicle docking | 5.06E-4 | 3.06E-2 |
| GO:0051235 | maintenance of location | 5.17E-4 | 3.12E-2 |
| GO:0051271 | negative regulation of cellular component movement | 5.23E-4 | 3.14E-2 |
| GO:0086001 | cardiac muscle cell action potential | 5.34E-4 | 3.2E-2 |
| GO:0051651 | maintenance of location in cell | 5.44E-4 | 3.25E-2 |
| GO:0099171 | presynaptic modulation of chemical synaptic transmission | 5.47E-4 | 3.25E-2 |
| GO:0021700 | developmental maturation | 5.72E-4 | 3.39E-2 |
| GO:0030258 | lipid modification | 5.78E-4 | 3.41E-2 |
| GO:0090066 | regulation of anatomical structure size | 5.82E-4 | 3.42E-2 |
| GO:0051058 | negative regulation of small GTPase mediated signal transduction | 5.82E-4 | 3.41E-2 |
| GO:0099633 | protein localization to postsynaptic specialization membrane | 5.85E-4 | 3.41E-2 |
| GO:0099645 | neurotransmitter receptor localization to postsynaptic specialization membrane | 5.85E-4 | 3.4E-2 |
| GO:1900452 | regulation of long-term synaptic depression | 5.85E-4 | 3.38E-2 |
| GO:0031915 | positive regulation of synaptic plasticity | 5.85E-4 | 3.37E-2 |
| GO:0051590 | positive regulation of neurotransmitter transport | 5.92E-4 | 3.4E-2 |
| GO:0006468 | protein phosphorylation | 6.13E-4 | 3.51E-2 |
| GO:0018210 | peptidyl-threonine modification | 6.13E-4 | 3.5E-2 |
| GO:0098828 | modulation of inhibitory postsynaptic potential | 6.17E-4 | 3.51E-2 |
| GO:0097061 | dendritic spine organization | 6.61E-4 | 3.74E-2 |
| GO:0006928 | movement of cell or subcellular component | 7.04E-4 | 3.97E-2 |
| GO:1905475 | regulation of protein localization to membrane | 7.21E-4 | 4.05E-2 |
| GO:0045737 | positive regulation of cyclin-dependent protein serine/threonine kinase activity | 8.1E-4 | 4.54E-2 |
| GO:0071625 | vocalization behavior | 8.16E-4 | 4.55E-2 |
| GO:0051241 | negative regulation of multicellular organismal process | 8.33E-4 | 4.63E-2 |
| GO:0032411 | positive regulation of transporter activity | 8.83E-4 | 4.89E-2 |
| GO:1902473 | regulation of protein localization to synapse | 9.07E-4 | 5.01E-2 |
| GO:0050775 | positive regulation of dendrite morphogenesis | 9.29E-4 | 5.11E-2 |
| GO:0048813 | dendrite morphogenesis | 9.29E-4 | 5.09E-2 |
| GO:0060401 | cytosolic calcium ion transport | 9.48E-4 | 5.18E-2 |
| GO:0007411 | axon guidance | 9.58E-4 | 5.22E-2 |
| GO:0048671 | negative regulation of collateral sprouting | 9.89E-4 | 5.36E-2 |
| GO:0035725 | sodium ion transmembrane transport | 9.9E-4 | 5.35E-2 |
| GO:0018209 | peptidyl-serine modification | 9.94E-4 | 5.36E-2 |
