# Supplementary material for: Repetitive transcranial magnetic stimulation activates glial cells and inhibits neurogenesis after pneumococcal meningitis
Source: PLoS One. 2020 Sep 11;15(9):e0232863. doi: 10.1371/journal.pone.0232863 (PMC7485822; doi:10.1371/journal.pone.0232863)
Supplement: S2 Table — (DOCX) [file pone.0232863.s008.docx]

Table S2. Overrepresented gene ontologies of upregulated genes after cTBS in the cortex.

| GO term | Description | P-value | FDR q-value |
| --- | --- | --- | --- |
| GO:0006412 | translation | 2.7E-23 | 4.17E-19 |
| GO:0043043 | peptide biosynthetic process | 3.28E-22 | 2.54E-18 |
| GO:0006518 | peptide metabolic process | 4.83E-21 | 2.49E-17 |
| GO:0043604 | amide biosynthetic process | 2.28E-19 | 8.82E-16 |
| GO:0043603 | cellular amide metabolic process | 1.78E-15 | 5.51E-12 |
| GO:0034641 | cellular nitrogen compound metabolic process | 5.56E-14 | 1.43E-10 |
| GO:0034645 | cellular macromolecule biosynthetic process | 6.63E-12 | 1.46E-8 |
| GO:0002181 | cytoplasmic translation | 1.11E-11 | 2.14E-8 |
| GO:0006396 | RNA processing | 1.26E-11 | 2.16E-8 |
| GO:0008152 | metabolic process | 1.49E-11 | 2.31E-8 |
| GO:0044237 | cellular metabolic process | 1.18E-10 | 1.66E-7 |
| GO:0009059 | macromolecule biosynthetic process | 2.62E-10 | 3.38E-7 |
| GO:1901566 | organonitrogen compound biosynthetic process | 2.9E-10 | 3.45E-7 |
| GO:0044271 | cellular nitrogen compound biosynthetic process | 7.21E-10 | 7.96E-7 |
| GO:0043170 | macromolecule metabolic process | 7.95E-10 | 8.19E-7 |
| GO:0006364 | rRNA processing | 1.38E-9 | 1.34E-6 |
| GO:0016072 | rRNA metabolic process | 1.33E-8 | 1.21E-5 |
| GO:0034470 | ncRNA processing | 2.4E-8 | 2.06E-5 |
| GO:0006807 | nitrogen compound metabolic process | 5.68E-8 | 4.62E-5 |
| GO:0000377 | RNA splicing, via transesterification reactions with bulged adenosine as nucleophile | 1E-7 | 7.76E-5 |
| GO:0000398 | mRNA splicing, via spliceosome | 1E-7 | 7.39E-5 |
| GO:0000375 | RNA splicing, via transesterification reactions | 1.12E-7 | 7.89E-5 |
| GO:0044260 | cellular macromolecule metabolic process | 1.31E-7 | 8.83E-5 |
| GO:0016070 | RNA metabolic process | 1.48E-7 | 9.54E-5 |
| GO:0071704 | organic substance metabolic process | 2.57E-7 | 1.59E-4 |
| GO:0034622 | cellular protein-containing complex assembly | 2.74E-7 | 1.63E-4 |
| GO:0044249 | cellular biosynthetic process | 6.98E-7 | 3.99E-4 |
| GO:0044238 | primary metabolic process | 1.03E-6 | 5.66E-4 |
| GO:0022618 | ribonucleoprotein complex assembly | 1.13E-6 | 6.03E-4 |
| GO:0090304 | nucleic acid metabolic process | 2.33E-6 | 1.2E-3 |
| GO:0034660 | ncRNA metabolic process | 2.78E-6 | 1.39E-3 |
| GO:0071826 | ribonucleoprotein complex subunit organization | 3.08E-6 | 1.49E-3 |
| GO:0008380 | RNA splicing | 3.84E-6 | 1.8E-3 |
| GO:0002376 | immune system process | 4.24E-6 | 1.93E-3 |
| GO:0006397 | mRNA processing | 5.33E-6 | 2.35E-3 |
| GO:0019538 | protein metabolic process | 6.46E-6 | 2.77E-3 |
| GO:0009058 | biosynthetic process | 6.76E-6 | 2.82E-3 |
| GO:0019884 | antigen processing and presentation of exogenous antigen | 6.79E-6 | 2.76E-3 |
| GO:0006139 | nucleobase-containing compound metabolic process | 9.62E-6 | 3.81E-3 |
| GO:0044085 | cellular component biogenesis | 1.08E-5 | 4.16E-3 |
| GO:0022613 | ribonucleoprotein complex biogenesis | 1.4E-5 | 5.28E-3 |
| GO:1901576 | organic substance biosynthetic process | 1.44E-5 | 5.29E-3 |
| GO:0044267 | cellular protein metabolic process | 1.76E-5 | 6.33E-3 |
| GO:0006950 | response to stress | 2.04E-5 | 7.17E-3 |
| GO:2000434 | regulation of protein neddylation | 2.44E-5 | 8.37E-3 |
| GO:0006725 | cellular aromatic compound metabolic process | 2.68E-5 | 8.99E-3 |
| GO:0032760 | positive regulation of tumor necrosis factor production | 3.02E-5 | 9.94E-3 |
| GO:0046483 | heterocycle metabolic process | 3.1E-5 | 9.98E-3 |
| GO:1903557 | positive regulation of tumor necrosis factor superfamily cytokine production | 3.38E-5 | 1.07E-2 |
| GO:0045321 | leukocyte activation | 3.44E-5 | 1.06E-2 |
| GO:0042221 | response to chemical | 3.53E-5 | 1.07E-2 |
| GO:0051716 | cellular response to stimulus | 3.57E-5 | 1.06E-2 |
| GO:0022900 | electron transport chain | 3.95E-5 | 1.15E-2 |
| GO:1904667 | negative regulation of ubiquitin protein ligase activity | 4.49E-5 | 1.28E-2 |
| GO:0033108 | mitochondrial respiratory chain complex assembly | 4.71E-5 | 1.32E-2 |
| GO:0016071 | mRNA metabolic process | 4.93E-5 | 1.36E-2 |
| GO:0009987 | cellular process | 5.97E-5 | 1.62E-2 |
| GO:0006979 | response to oxidative stress | 6.4E-5 | 1.7E-2 |
| GO:0050865 | regulation of cell activation | 6.94E-5 | 1.82E-2 |
| GO:0043933 | protein-containing complex subunit organization | 7.91E-5 | 2.04E-2 |
| GO:0010257 | NADH dehydrogenase complex assembly | 9.64E-5 | 2.44E-2 |
| GO:0032981 | mitochondrial respiratory chain complex I assembly | 9.64E-5 | 2.4E-2 |
| GO:0002694 | regulation of leukocyte activation | 9.68E-5 | 2.37E-2 |
| GO:0002252 | immune effector process | 1.21E-4 | 2.93E-2 |
| GO:0032101 | regulation of response to external stimulus | 1.3E-4 | 3.09E-2 |
| GO:0030193 | regulation of blood coagulation | 1.3E-4 | 3.06E-2 |
| GO:1900046 | regulation of hemostasis | 1.47E-4 | 3.38E-2 |
| GO:0051249 | regulation of lymphocyte activation | 1.57E-4 | 3.57E-2 |
| GO:0030889 | negative regulation of B cell proliferation | 1.68E-4 | 3.76E-2 |
| GO:0002683 | negative regulation of immune system process | 1.7E-4 | 3.75E-2 |
| GO:0001775 | cell activation | 1.84E-4 | 4.02E-2 |
| GO:0050818 | regulation of coagulation | 2.05E-4 | 4.4E-2 |
| GO:0034248 | regulation of cellular amide metabolic process | 2.07E-4 | 4.39E-2 |
| GO:0002431 | Fc receptor mediated stimulatory signaling pathway | 2.33E-4 | 4.87E-2 |
| GO:0002765 | immune response-inhibiting signal transduction | 2.33E-4 | 4.81E-2 |
| GO:1901360 | organic cyclic compound metabolic process | 2.35E-4 | 4.77E-2 |
| GO:1901564 | organonitrogen compound metabolic process | 2.36E-4 | 4.75E-2 |
| GO:0019882 | antigen processing and presentation | 2.54E-4 | 5.03E-2 |
| GO:0022904 | respiratory electron transport chain | 2.55E-4 | 5E-2 |
| GO:0045454 | cell redox homeostasis | 2.55E-4 | 4.94E-2 |
| GO:0002682 | regulation of immune system process | 2.79E-4 | 5.32E-2 |
| GO:0002478 | antigen processing and presentation of exogenous peptide antigen | 2.8E-4 | 5.28E-2 |
| GO:0045730 | respiratory burst | 2.89E-4 | 5.38E-2 |
| GO:0050896 | response to stimulus | 2.94E-4 | 5.4E-2 |
| GO:0046649 | lymphocyte activation | 3.23E-4 | 5.88E-2 |
| GO:0000302 | response to reactive oxygen species | 3.34E-4 | 6.01E-2 |
| GO:0050727 | regulation of inflammatory response | 3.37E-4 | 5.99E-2 |
| GO:0022411 | cellular component disassembly | 3.51E-4 | 6.17E-2 |
| GO:0010499 | proteasomal ubiquitin-independent protein catabolic process | 3.54E-4 | 6.15E-2 |
| GO:0045087 | innate immune response | 3.67E-4 | 6.31E-2 |
| GO:0009719 | response to endogenous stimulus | 3.99E-4 | 6.77E-2 |
| GO:0050776 | regulation of immune response | 4.05E-4 | 6.8E-2 |
| GO:0042542 | response to hydrogen peroxide | 4.09E-4 | 6.8E-2 |
| GO:0050670 | regulation of lymphocyte proliferation | 4.31E-4 | 7.08E-2 |
| GO:0016064 | immunoglobulin mediated immune response | 4.42E-4 | 7.19E-2 |
| GO:0002449 | lymphocyte mediated immunity | 4.55E-4 | 7.32E-2 |
| GO:0030888 | regulation of B cell proliferation | 4.55E-4 | 7.25E-2 |
| GO:0097421 | liver regeneration | 4.55E-4 | 7.17E-2 |
| GO:0032944 | regulation of mononuclear cell proliferation | 4.78E-4 | 7.47E-2 |
| GO:0009636 | response to toxic substance | 5.06E-4 | 7.83E-2 |
| GO:0002250 | adaptive immune response | 5.35E-4 | 8.18E-2 |
| GO:0071236 | cellular response to antibiotic | 5.66E-4 | 8.57E-2 |
| GO:0006417 | regulation of translation | 5.82E-4 | 8.73E-2 |
| GO:0010941 | regulation of cell death | 5.99E-4 | 8.89E-2 |
| GO:0043067 | regulation of programmed cell death | 6.01E-4 | 8.84E-2 |
| GO:0046907 | intracellular transport | 6.13E-4 | 8.94E-2 |
| GO:0042273 | ribosomal large subunit biogenesis | 6.65E-4 | 9.6E-2 |
| GO:0042981 | regulation of apoptotic process | 7.02E-4 | 1E-1 |
| GO:0070663 | regulation of leukocyte proliferation | 7.16E-4 | 1.02E-1 |
| GO:0032755 | positive regulation of interleukin-6 production | 7.28E-4 | 1.02E-1 |
| GO:0006952 | defense response | 7.42E-4 | 1.03E-1 |
| GO:1904666 | regulation of ubiquitin protein ligase activity | 7.44E-4 | 1.03E-1 |
| GO:0046677 | response to antibiotic | 7.54E-4 | 1.03E-1 |
| GO:0060369 | positive regulation of Fc receptor mediated stimulatory signaling pathway | 7.82E-4 | 1.06E-1 |
| GO:0050866 | negative regulation of cell activation | 7.91E-4 | 1.06E-1 |
| GO:0019724 | B cell mediated immunity | 8.04E-4 | 1.07E-1 |
| GO:0006955 | immune response | 8.19E-4 | 1.08E-1 |
| GO:0065003 | protein-containing complex assembly | 8.41E-4 | 1.1E-1 |
| GO:0002774 | Fc receptor mediated inhibitory signaling pathway | 8.43E-4 | 1.09E-1 |
| GO:0052565 | response to defense-related host nitric oxide production | 8.43E-4 | 1.09E-1 |
| GO:0052551 | response to defense-related nitric oxide production by other organism involved in symbiotic interaction | 8.43E-4 | 1.08E-1 |
| GO:0018916 | nitrobenzene metabolic process | 8.43E-4 | 1.07E-1 |
| GO:0032917 | polyamine acetylation | 8.43E-4 | 1.06E-1 |
| GO:0032918 | spermidine acetylation | 8.43E-4 | 1.05E-1 |
| GO:0032919 | spermine acetylation | 8.43E-4 | 1.04E-1 |
| GO:2000435 | negative regulation of protein neddylation | 8.43E-4 | 1.03E-1 |
| GO:0007113 | endomitotic cell cycle | 8.43E-4 | 1.03E-1 |
| GO:1905663 | positive regulation of telomerase RNA reverse transcriptase activity | 8.43E-4 | 1.02E-1 |
| GO:0032680 | regulation of tumor necrosis factor production | 8.43E-4 | 1.01E-1 |
| GO:0051250 | negative regulation of lymphocyte activation | 8.43E-4 | 1E-1 |
| GO:0010033 | response to organic substance | 8.7E-4 | 1.03E-1 |
| GO:0035690 | cellular response to drug | 8.77E-4 | 1.03E-1 |
| GO:0002695 | negative regulation of leukocyte activation | 9.03E-4 | 1.05E-1 |
| GO:1903555 | regulation of tumor necrosis factor superfamily cytokine production | 9.54E-4 | 1.1E-1 |
| GO:0000027 | ribosomal large subunit assembly | 9.63E-4 | 1.1E-1 |
| GO:0006120 | mitochondrial electron transport, NADH to ubiquinone | 9.69E-4 | 1.1E-1 |
