# Supplementary material for: Repetitive transcranial magnetic stimulation activates glial cells and inhibits neurogenesis after pneumococcal meningitis
Source: PLoS One. 2020 Sep 11;15(9):e0232863. doi: 10.1371/journal.pone.0232863 (PMC7485822; doi:10.1371/journal.pone.0232863)
Supplement: S3 Table — (DOCX) [file pone.0232863.s009.docx]

Table S3. Overrepresented gene ontologies of downregulated genes after cTBS in the hippocampus.

| GO term | Description | P-value | FDR q-value |
| --- | --- | --- | --- |
| GO:0051253 | negative regulation of RNA metabolic process | 2.39E-7 | 3.69E-3 |
| GO:0045934 | negative regulation of nucleobase-containing compound metabolic process | 9.54E-7 | 7.38E-3 |
| GO:0045892 | negative regulation of transcription, DNA-templated | 2.75E-6 | 1.42E-2 |
| GO:1903507 | negative regulation of nucleic acid-templated transcription | 4.46E-6 | 1.72E-2 |
| GO:1902679 | negative regulation of RNA biosynthetic process | 4.63E-6 | 1.43E-2 |
| GO:0031324 | negative regulation of cellular metabolic process | 5.45E-6 | 1.4E-2 |
| GO:0051172 | negative regulation of nitrogen compound metabolic process | 1.02E-5 | 2.26E-2 |
| GO:0000122 | negative regulation of transcription by RNA polymerase II | 1.3E-5 | 2.51E-2 |
| GO:0010605 | negative regulation of macromolecule metabolic process | 1.46E-5 | 2.51E-2 |
| GO:0003002 | regionalization | 1.76E-5 | 2.72E-2 |
| GO:2000113 | negative regulation of cellular macromolecule biosynthetic process | 2.33E-5 | 3.28E-2 |
| GO:0048523 | negative regulation of cellular process | 3.79E-5 | 4.88E-2 |
| GO:0048592 | eye morphogenesis | 3.89E-5 | 4.63E-2 |
| GO:0010558 | negative regulation of macromolecule biosynthetic process | 5.29E-5 | 5.84E-2 |
| GO:0048562 | embryonic organ morphogenesis | 5.43E-5 | 5.59E-2 |
| GO:0060412 | ventricular septum morphogenesis | 7.35E-5 | 7.11E-2 |
| GO:0009892 | negative regulation of metabolic process | 8.1E-5 | 7.37E-2 |
| GO:0010629 | negative regulation of gene expression | 8.77E-5 | 7.53E-2 |
| GO:0090596 | sensory organ morphogenesis | 9.1E-5 | 7.41E-2 |
| GO:0017015 | regulation of transforming growth factor beta receptor signaling pathway | 1.07E-4 | 8.26E-2 |
| GO:0031327 | negative regulation of cellular biosynthetic process | 1.15E-4 | 8.44E-2 |
| GO:1903844 | regulation of cellular response to transforming growth factor beta stimulus | 1.22E-4 | 8.56E-2 |
| GO:0009890 | negative regulation of biosynthetic process | 1.74E-4 | 1.17E-1 |
| GO:0048519 | negative regulation of biological process | 1.8E-4 | 1.16E-1 |
| GO:0030511 | positive regulation of transforming growth factor beta receptor signaling pathway | 1.94E-4 | 1.2E-1 |
| GO:1903846 | positive regulation of cellular response to transforming growth factor beta stimulus | 1.94E-4 | 1.16E-1 |
| GO:0007389 | pattern specification process | 1.96E-4 | 1.12E-1 |
| GO:1902369 | negative regulation of RNA catabolic process | 2.45E-4 | 1.35E-1 |
| GO:0031323 | regulation of cellular metabolic process | 2.54E-4 | 1.36E-1 |
| GO:1990416 | cellular response to brain-derived neurotrophic factor stimulus | 2.9E-4 | 1.49E-1 |
| GO:0033962 | cytoplasmic mRNA processing body assembly | 2.9E-4 | 1.45E-1 |
| GO:0090287 | regulation of cellular response to growth factor stimulus | 4.78E-4 | 2.31E-1 |
| GO:0001837 | epithelial to mesenchymal transition | 4.92E-4 | 2.31E-1 |
| GO:0048640 | negative regulation of developmental growth | 5.71E-4 | 2.6E-1 |
| GO:0097306 | cellular response to alcohol | 5.71E-4 | 2.52E-1 |
| GO:0043487 | regulation of RNA stability | 6.11E-4 | 2.63E-1 |
| GO:0060325 | face morphogenesis | 6.29E-4 | 2.63E-1 |
| GO:0050794 | regulation of cellular process | 6.72E-4 | 2.74E-1 |
| GO:0030182 | neuron differentiation | 6.89E-4 | 2.73E-1 |
| GO:0090092 | regulation of transmembrane receptor protein serine/threonine kinase signaling pathway | 6.92E-4 | 2.67E-1 |
| GO:0006357 | regulation of transcription by RNA polymerase II | 6.97E-4 | 2.63E-1 |
| GO:0048596 | embryonic camera-type eye morphogenesis | 7.14E-4 | 2.63E-1 |
| GO:0045926 | negative regulation of growth | 7.35E-4 | 2.64E-1 |
| GO:0001501 | skeletal system development | 7.53E-4 | 2.65E-1 |
| GO:0055026 | negative regulation of cardiac muscle tissue development | 7.79E-4 | 2.68E-1 |
| GO:0060255 | regulation of macromolecule metabolic process | 9E-4 | 3.03E-1 |
| GO:0009887 | animal organ morphogenesis | 9.33E-4 | 3.07E-1 |
| GO:0043489 | RNA stabilization | 9.53E-4 | 3.07E-1 |
