# Supplementary material for: Repetitive transcranial magnetic stimulation activates glial cells and inhibits neurogenesis after pneumococcal meningitis
Source: PLoS One. 2020 Sep 11;15(9):e0232863. doi: 10.1371/journal.pone.0232863 (PMC7485822; doi:10.1371/journal.pone.0232863)
Supplement: S4 Table — (DOCX) [file pone.0232863.s010.docx]

Table S4. Overrepresented gene ontologies in upregulated genes after cTBS in the hippocampus.

| GO term | Description | P-value | FDR q-value |
| --- | --- | --- | --- |
| GO:0006518 | peptide metabolic process | 2.55E-18 | 3.95E-14 |
| GO:0006412 | translation | 1.29E-16 | 1E-12 |
| GO:0043043 | peptide biosynthetic process | 6.42E-16 | 3.31E-12 |
| GO:0043603 | cellular amide metabolic process | 6.86E-14 | 2.65E-10 |
| GO:0043604 | amide biosynthetic process | 8.14E-13 | 2.52E-9 |
| GO:0034622 | cellular protein-containing complex assembly | 3.3E-8 | 8.51E-5 |
| GO:0034645 | cellular macromolecule biosynthetic process | 1.08E-7 | 2.39E-4 |
| GO:0009059 | macromolecule biosynthetic process | 2.1E-7 | 4.07E-4 |
| GO:0022618 | ribonucleoprotein complex assembly | 3.35E-7 | 5.76E-4 |
| GO:0071826 | ribonucleoprotein complex subunit organization | 7.93E-7 | 1.23E-3 |
| GO:1901566 | organonitrogen compound biosynthetic process | 1.02E-6 | 1.43E-3 |
| GO:0002181 | cytoplasmic translation | 1.93E-6 | 2.48E-3 |
| GO:0000027 | ribosomal large subunit assembly | 5.59E-6 | 6.64E-3 |
| GO:0044267 | cellular protein metabolic process | 6.31E-6 | 6.97E-3 |
| GO:0008152 | metabolic process | 7.8E-6 | 8.04E-3 |
| GO:0044271 | cellular nitrogen compound biosynthetic process | 1.08E-5 | 1.05E-2 |
| GO:0055114 | oxidation-reduction process | 1.2E-5 | 1.09E-2 |
| GO:0043933 | protein-containing complex subunit organization | 1.33E-5 | 1.14E-2 |
| GO:0000028 | ribosomal small subunit assembly | 1.44E-5 | 1.17E-2 |
| GO:0044237 | cellular metabolic process | 1.75E-5 | 1.35E-2 |
| GO:1900426 | positive regulation of defense response to bacterium | 2.65E-5 | 1.95E-2 |
| GO:0065003 | protein-containing complex assembly | 3.41E-5 | 2.39E-2 |
| GO:0110095 | cellular detoxification of aldehyde | 5.02E-5 | 3.38E-2 |
| GO:0033108 | mitochondrial respiratory chain complex assembly | 6.86E-5 | 4.42E-2 |
| GO:0044260 | cellular macromolecule metabolic process | 8.95E-5 | 5.54E-2 |
| GO:0019538 | protein metabolic process | 9.13E-5 | 5.43E-2 |
| GO:0031647 | regulation of protein stability | 1.02E-4 | 5.85E-2 |
| GO:0070301 | cellular response to hydrogen peroxide | 1.11E-4 | 6.15E-2 |
| GO:0044249 | cellular biosynthetic process | 1.28E-4 | 6.8E-2 |
| GO:1901576 | organic substance biosynthetic process | 1.45E-4 | 7.47E-2 |
| GO:0009058 | biosynthetic process | 1.52E-4 | 7.59E-2 |
| GO:1901564 | organonitrogen compound metabolic process | 1.55E-4 | 7.49E-2 |
| GO:1900424 | regulation of defense response to bacterium | 2.23E-4 | 1.05E-1 |
| GO:2001198 | regulation of dendritic cell differentiation | 2.7E-4 | 1.23E-1 |
| GO:0018916 | nitrobenzene metabolic process | 2.99E-4 | 1.32E-1 |
| GO:0043902 | positive regulation of multi-organism process | 3.94E-4 | 1.69E-1 |
| GO:0032464 | positive regulation of protein homooligomerization | 4.01E-4 | 1.67E-1 |
| GO:1990748 | cellular detoxification | 4.02E-4 | 1.64E-1 |
| GO:0010257 | NADH dehydrogenase complex assembly | 4.74E-4 | 1.88E-1 |
| GO:0032981 | mitochondrial respiratory chain complex I assembly | 4.74E-4 | 1.83E-1 |
| GO:0042178 | xenobiotic catabolic process | 5.65E-4 | 2.13E-1 |
| GO:0050792 | regulation of viral process | 6.12E-4 | 2.25E-1 |
| GO:0034641 | cellular nitrogen compound metabolic process | 6.63E-4 | 2.39E-1 |
| GO:0006457 | protein folding | 6.75E-4 | 2.37E-1 |
| GO:0022607 | cellular component assembly | 7.61E-4 | 2.62E-1 |
| GO:0061635 | regulation of protein complex stability | 7.67E-4 | 2.58E-1 |
| GO:0098754 | detoxification | 7.79E-4 | 2.56E-1 |
| GO:0007034 | vacuolar transport | 7.81E-4 | 2.52E-1 |
| GO:0009056 | catabolic process | 8.12E-4 | 2.56E-1 |
| GO:2001199 | negative regulation of dendritic cell differentiation | 8.88E-4 | 2.75E-1 |
| GO:0071704 | organic substance metabolic process | 9.11E-4 | 2.76E-1 |
