# Supplementary material for: Repetitive transcranial magnetic stimulation activates glial cells and inhibits neurogenesis after pneumococcal meningitis
Source: PLoS One. 2020 Sep 11;15(9):e0232863. doi: 10.1371/journal.pone.0232863 (PMC7485822; doi:10.1371/journal.pone.0232863)
Supplement: S5 Table — (DOCX) [file pone.0232863.s011.docx]

Table S5. Overrepresented gene ontologies in downregulated genes after iTBS in the cortex.

| GO term | Description | P-value | FDR q-value |
| --- | --- | --- | --- |
| GO:0050804 | modulation of chemical synaptic transmission | 4.26E-9 | 6.58E-5 |
| GO:0099177 | regulation of trans-synaptic signaling | 4.36E-9 | 3.37E-5 |
| GO:0035176 | social behavior | 1.81E-6 | 9.35E-3 |
| GO:0051703 | intraspecies interaction between organisms | 1.81E-6 | 7.01E-3 |
| GO:0007612 | learning | 2.21E-6 | 6.83E-3 |
| GO:0048167 | regulation of synaptic plasticity | 2.92E-6 | 7.53E-3 |
| GO:0099003 | vesicle-mediated transport in synapse | 4.15E-6 | 9.15E-3 |
| GO:0051234 | establishment of localization | 4.34E-6 | 8.39E-3 |
| GO:0051649 | establishment of localization in cell | 5.68E-6 | 9.75E-3 |
| GO:0051049 | regulation of transport | 5.72E-6 | 8.84E-3 |
| GO:0006836 | neurotransmitter transport | 6.91E-6 | 9.71E-3 |
| GO:0051705 | multi-organism behavior | 7.6E-6 | 9.79E-3 |
| GO:0006810 | transport | 7.7E-6 | 9.16E-3 |
| GO:0007611 | learning or memory | 8.47E-6 | 9.35E-3 |
| GO:0051641 | cellular localization | 8.87E-6 | 9.13E-3 |
| GO:0017158 | regulation of calcium ion-dependent exocytosis | 9.34E-6 | 9.03E-3 |
| GO:0050806 | positive regulation of synaptic transmission | 1.3E-5 | 1.18E-2 |
| GO:0050890 | cognition | 1.77E-5 | 1.52E-2 |
| GO:0051179 | localization | 1.8E-5 | 1.47E-2 |
| GO:0007215 | glutamate receptor signaling pathway | 2.59E-5 | 2E-2 |
| GO:0032879 | regulation of localization | 3.11E-5 | 2.29E-2 |
| GO:0016082 | synaptic vesicle priming | 3.46E-5 | 2.43E-2 |
| GO:0060125 | negative regulation of growth hormone secretion | 3.81E-5 | 2.56E-2 |
| GO:1903305 | regulation of regulated secretory pathway | 4.53E-5 | 2.91E-2 |
| GO:0035418 | protein localization to synapse | 4.79E-5 | 2.96E-2 |
| GO:0060341 | regulation of cellular localization | 6.11E-5 | 3.63E-2 |
| GO:0016192 | vesicle-mediated transport | 6.59E-5 | 3.77E-2 |
| GO:1902473 | regulation of protein localization to synapse | 8.46E-5 | 4.67E-2 |
| GO:0051648 | vesicle localization | 8.77E-5 | 4.68E-2 |
| GO:0060627 | regulation of vesicle-mediated transport | 1.06E-4 | 5.44E-2 |
| GO:0099643 | signal release from synapse | 1.15E-4 | 5.75E-2 |
| GO:0046928 | regulation of neurotransmitter secretion | 1.17E-4 | 5.67E-2 |
| GO:0035235 | ionotropic glutamate receptor signaling pathway | 1.36E-4 | 6.35E-2 |
| GO:0051966 | regulation of synaptic transmission, glutamatergic | 1.36E-4 | 6.17E-2 |
| GO:0140029 | exocytic process | 1.36E-4 | 5.99E-2 |
| GO:0097479 | synaptic vesicle localization | 1.51E-4 | 6.47E-2 |
| GO:0007613 | memory | 1.59E-4 | 6.65E-2 |
| GO:0098693 | regulation of synaptic vesicle cycle | 1.65E-4 | 6.69E-2 |
| GO:0023061 | signal release | 1.65E-4 | 6.52E-2 |
| GO:0065008 | regulation of biological quality | 1.69E-4 | 6.53E-2 |
| GO:0006941 | striated muscle contraction | 1.76E-4 | 6.62E-2 |
| GO:0099011 | neuronal dense core vesicle exocytosis | 1.89E-4 | 6.96E-2 |
| GO:0099525 | presynaptic dense core vesicle exocytosis | 1.89E-4 | 6.8E-2 |
| GO:0017157 | regulation of exocytosis | 1.95E-4 | 6.85E-2 |
| GO:0045956 | positive regulation of calcium ion-dependent exocytosis | 2.03E-4 | 6.98E-2 |
| GO:1900454 | positive regulation of long-term synaptic depression | 2.64E-4 | 8.88E-2 |
| GO:1990504 | dense core granule exocytosis | 2.64E-4 | 8.69E-2 |
| GO:0086036 | regulation of cardiac muscle cell membrane potential | 2.64E-4 | 8.51E-2 |
| GO:0051968 | positive regulation of synaptic transmission, glutamatergic | 3.14E-4 | 9.91E-2 |
| GO:1903530 | regulation of secretion by cell | 3.28E-4 | 1.01E-1 |
| GO:0099132 | ATP hydrolysis coupled cation transmembrane transport | 3.4E-4 | 1.03E-1 |
| GO:0051640 | organelle localization | 3.64E-4 | 1.08E-1 |
| GO:0051588 | regulation of neurotransmitter transport | 3.96E-4 | 1.15E-1 |
| GO:0097120 | receptor localization to synapse | 4.27E-4 | 1.22E-1 |
| GO:0007269 | neurotransmitter secretion | 4.27E-4 | 1.2E-1 |
| GO:0007616 | long-term memory | 4.58E-4 | 1.27E-1 |
| GO:2000300 | regulation of synaptic vesicle exocytosis | 4.91E-4 | 1.33E-1 |
| GO:0007156 | homophilic cell adhesion via plasma membrane adhesion molecules | 4.91E-4 | 1.31E-1 |
| GO:0008306 | associative learning | 5.09E-4 | 1.33E-1 |
| GO:0051046 | regulation of secretion | 5.49E-4 | 1.41E-1 |
| GO:1902803 | regulation of synaptic vesicle transport | 5.68E-4 | 1.44E-1 |
| GO:0007420 | brain development | 6.08E-4 | 1.52E-1 |
| GO:0060048 | cardiac muscle contraction | 6.39E-4 | 1.57E-1 |
| GO:0051650 | establishment of vesicle localization | 7.22E-4 | 1.74E-1 |
| GO:0055082 | cellular chemical homeostasis | 7.44E-4 | 1.77E-1 |
| GO:0042391 | regulation of membrane potential | 7.8E-4 | 1.83E-1 |
| GO:0034765 | regulation of ion transmembrane transport | 9.06E-4 | 2.09E-1 |
| GO:0070296 | sarcoplasmic reticulum calcium ion transport | 9.68E-4 | 2.2E-1 |
