# Supplementary material for: Repetitive transcranial magnetic stimulation activates glial cells and inhibits neurogenesis after pneumococcal meningitis
Source: PLoS One. 2020 Sep 11;15(9):e0232863. doi: 10.1371/journal.pone.0232863 (PMC7485822; doi:10.1371/journal.pone.0232863)
Supplement: S6 Table — (DOCX) [file pone.0232863.s012.docx]

Table S6. Overrepresented gene ontologies in upregulated genes after iTBS in the cortex.

| GO term | Description | P-value | FDR q-value |
| --- | --- | --- | --- |
| GO:0006412 | translation | 2.01E-4 | 1E0 |
| GO:0043043 | peptide biosynthetic process | 2.42E-4 | 1E0 |
| GO:0034443 | negative regulation of lipoprotein oxidation | 6.12E-4 | 1E0 |
| GO:1904247 | positive regulation of polynucleotide adenylyltransferase activity | 6.12E-4 | 1E0 |
| GO:0060588 | negative regulation of lipoprotein lipid oxidation | 6.12E-4 | 1E0 |
| GO:0043604 | amide biosynthetic process | 7.01E-4 | 1E0 |
| GO:0006518 | peptide metabolic process | 7.9E-4 | 1E0 |
