# Supplementary material for: Repetitive transcranial magnetic stimulation activates glial cells and inhibits neurogenesis after pneumococcal meningitis
Source: PLoS One. 2020 Sep 11;15(9):e0232863. doi: 10.1371/journal.pone.0232863 (PMC7485822; doi:10.1371/journal.pone.0232863)
Supplement: S2 Fig — Significantly overrepresented gene ontologies are highlighted in grey, with darker colour representing more significant p-values (see scale). In the cortex, cTBS induced a significant upregulation of processes involved 1.) translation, 2.) RNA processing, 3.) ribonucleoprotein complex assembly, 4.) response to stimuli, 5.) immune system regulation and 6.) regulation of TNF production. (PDF) [file pone.0232863.s002.pdf]

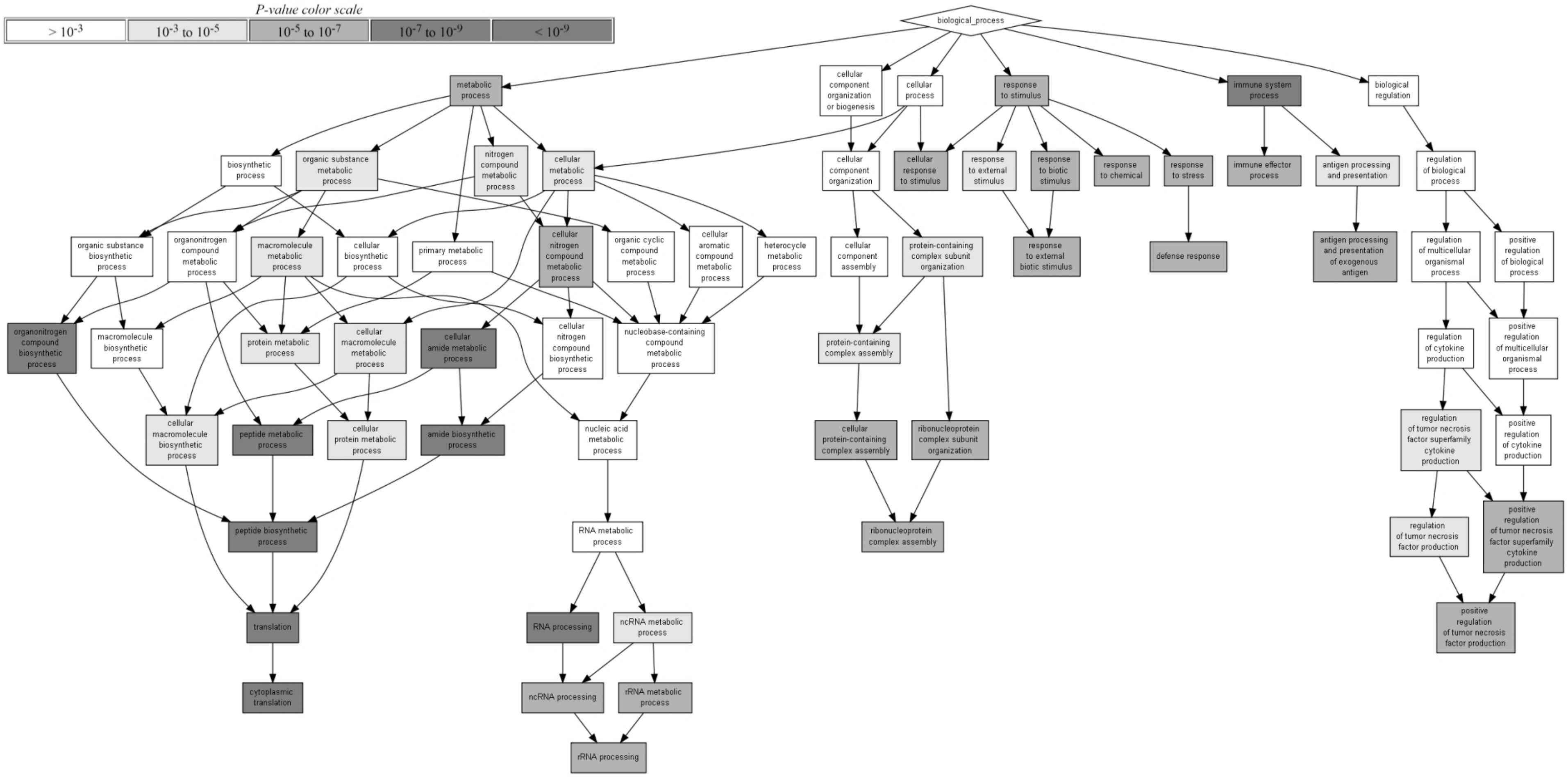

Figure S2. Overrepresented gene ontologies in upregulated genes upon cTBS in the cortex. Significantly overrepresented gene ontologies are highlighted in grey, with darker colour representing more significant p-values (see scale). In the cortex, cTBS induced a significant upregulation of processes involved 1.) translation, 2.) RNA processing, 3.) ribonucleoprotein complex assembly, 4.) response to stimuli, 5.) immune system regulation and 6.) regulation of TNF production.
