# Supplementary material for: Repetitive transcranial magnetic stimulation activates glial cells and inhibits neurogenesis after pneumococcal meningitis
Source: PLoS One. 2020 Sep 11;15(9):e0232863. doi: 10.1371/journal.pone.0232863 (PMC7485822; doi:10.1371/journal.pone.0232863)
Supplement: S3 Fig — Significantly overrepresented gene ontologies are highlighted in grey, with darker colour representing more significant p-values (see scale). In the cortex, cTBS induced a significant downregulation of processes involved 1.) regulation of transmembrane transport, 2.) regulation of developmental processes including neurogenesis and axonogenesis, 3.) regulation of synaptic plasticity and excitatory postsynaptic potentials, 4.) neuron projection morphogenesis, 5.) transmembrane ion transport and 6.) nervous system processes such as cognition, learning and memory. (PDF) [file pone.0232863.s003.pdf]

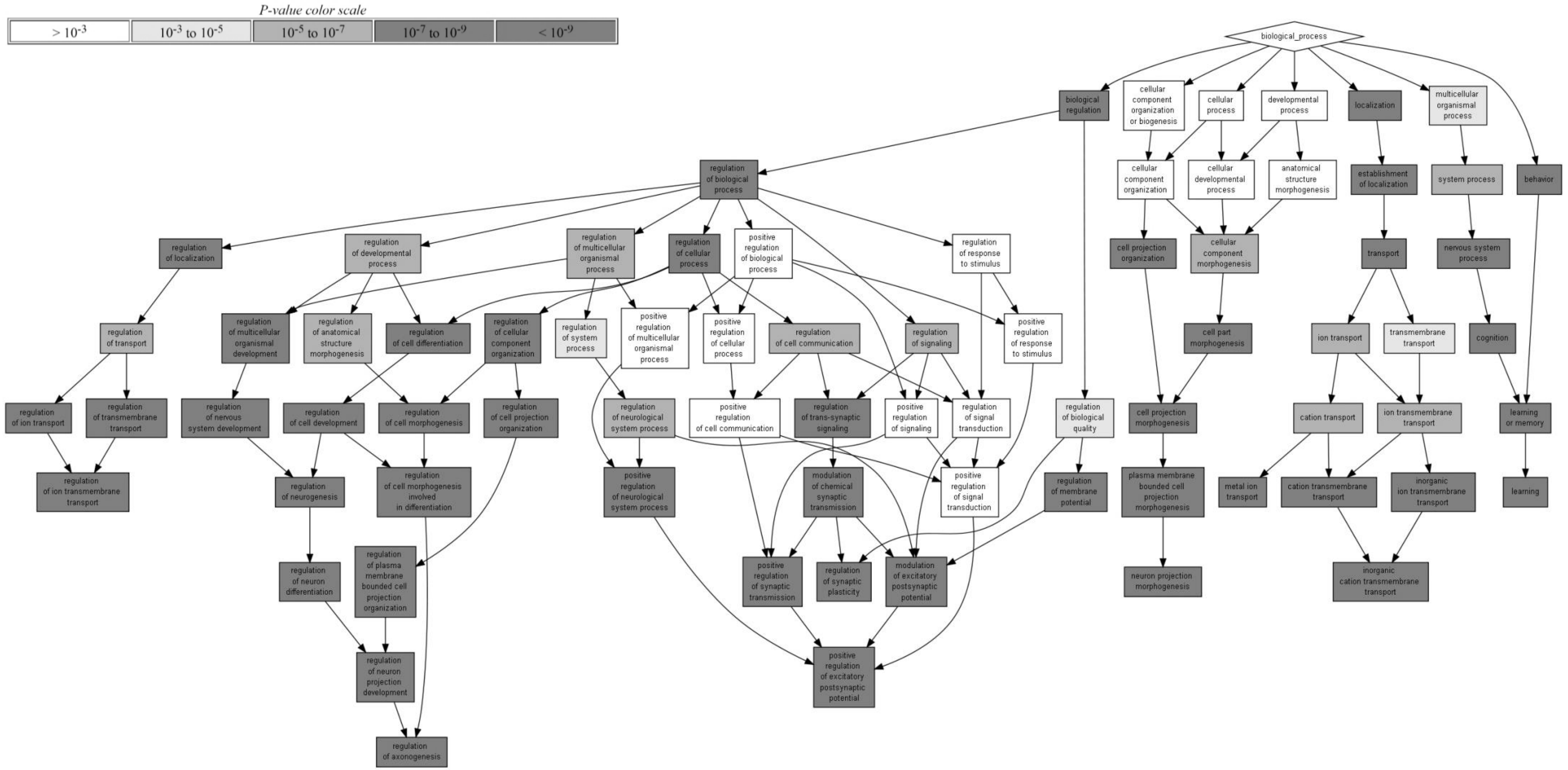

Figure S3. Overrepresented gene ontologies in downregulated genes upon cTBS in the cortex. Significantly overrepresented gene ontologies are highlighted in grey, with darker colour representing more significant p-values (see scale). In the cortex, cTBS induced a significant downregulation of processes involved 1.) regulation of transmembrane transport, 2.) regulation of developmental processes including neurogenesis and axonogenesis, 3.) regulation of synaptic plasticity and excitatory postsynaptic potentials, 4.) neuron projection morphogenesis, 5.) transmembrane ion transport and 6.) nervous system processes such as cognition, learning and memory.
