# Supplementary material for: Repetitive transcranial magnetic stimulation activates glial cells and inhibits neurogenesis after pneumococcal meningitis
Source: PLoS One. 2020 Sep 11;15(9):e0232863. doi: 10.1371/journal.pone.0232863 (PMC7485822; doi:10.1371/journal.pone.0232863)
Supplement: S5 Fig — Significantly overrepresented gene ontologies are in grey, with darker colour representing more significant p-values (see scale). In the cortex, cTBS induced a significant downregulation of processes involved 1.) negative regulators of transcription and translation; 2.) cellular response to brain-derived neurotrophic factor (BDNF); 3.) regulation of transforming growth factor beta receptor signalling pathway; and 4.) cellular development processes with decreased neuron differentiation. (PDF) [file pone.0232863.s005.pdf]

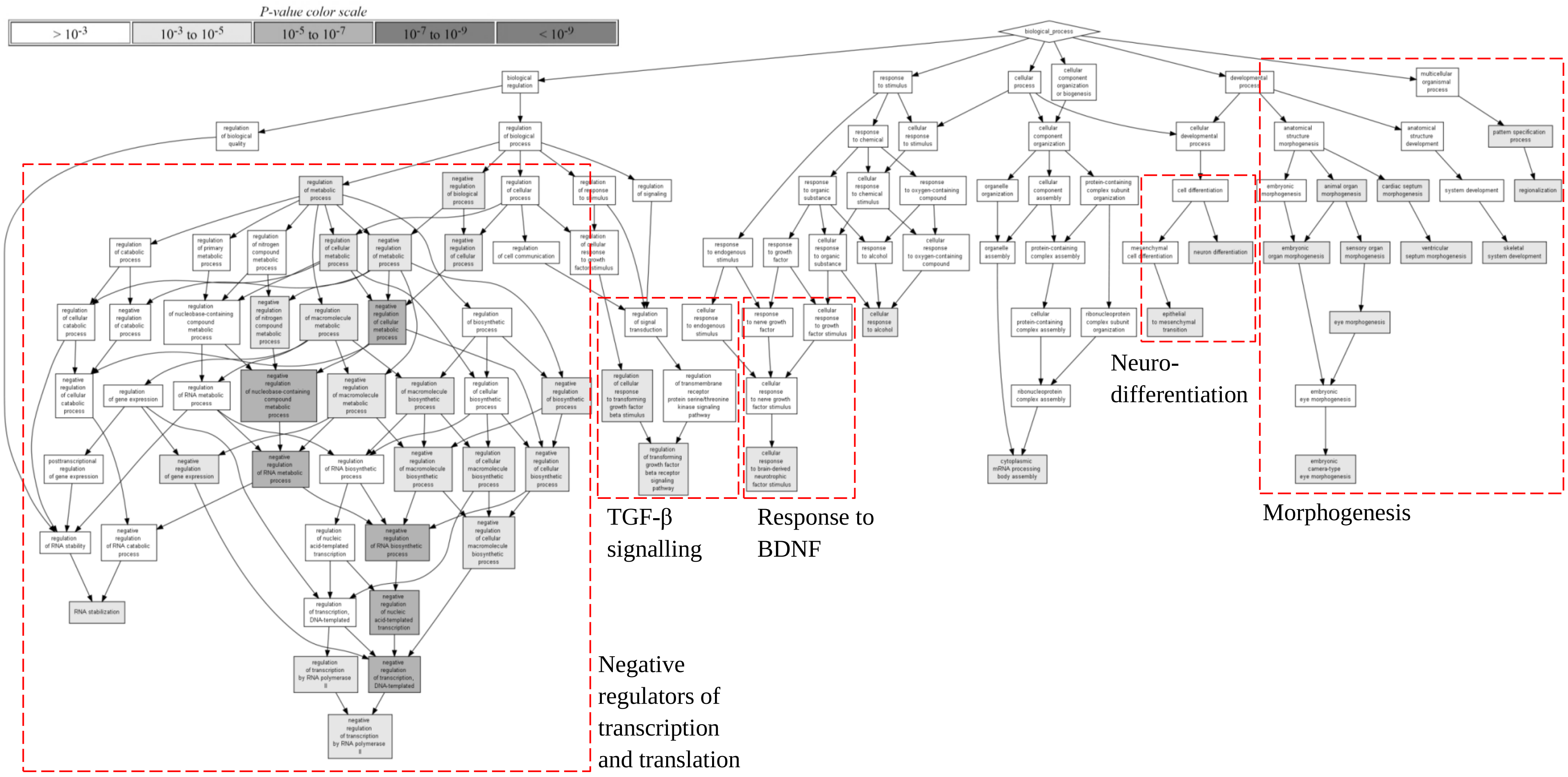

Figure S5. Overrepresented gene ontologies in downregualted genes upon cTBS in the hippocampus. Significantly overrepresented gene ontologies are in grey, with darker colour representing more significant p-values (see scale). In the cortex, cTBS induced a significant downregulation of processes involved 1.) negative regulators of transcription and translation; 2.) cellular response to brain-derived neurotrophic factor (BDNF); 3.) regulation of transforming growth factor beta receptor signalling pathway; and 4.) cellular development processes with decreased neuron differentiation.
